# Epidemiology, treatments, and related biomarkers of locally advanced or metastatic urothelial carcinoma in Chinese population: A scoping review

**DOI:** 10.1002/cam4.6112

**Published:** 2023-06-30

**Authors:** Wang He, Changhao Chen, Tianxin Lin, Qian Xu, Chong Ye, Jieyi Du, Jian Huang

**Affiliations:** ^1^ Department of Urology Sun Yat‐sen Memorial Hospital, Sun Yat‐sen University Guangzhou China; ^2^ Guangdong Provincial Key Laboratory of Malignant Tumor Epigenetics and Gene Regulation Sun Yat‐sen Memorial Hospital, State Key Laboratory of Oncology in South China Guangzhou China; ^3^ Xi'an Janssen Pharmaceutical Ltd. Beijing China

**Keywords:** biomarker, bladder cancer, epidemiology, la/mUC, scoping review, treatment

## Abstract

**Objective:**

Bladder cancer is the 13th most common cancer in China with the predominant histologic type being urothelial carcinoma (UC). Locally advanced and metastatic (la/m) UC accounts for 12% of UC and the five‐year survival rate is only 39.4%, imposing a significant disease and economic burden on the patients. The aim of this scoping review is to synthesize existing evidence of epidemiology, the landscape of treatment options and associated efficacy and safety profiles, as well as treatment‐related biomarkers among Chinese la/mUC patients.

**Methods:**

A systematic search was conducted on five databases (PubMed, Web of Science, Embase, Wanfang, and CNKI) from January 2011 to March 2022 based on the scoping review criteria in accordance with the guidelines of the Preferred Reporting Items for Systematic Reviews and Meta‐Analysis Extension for Scoping Reviews.

**Results:**

A total of 6211 records were identified, and further review resulted in 41 relevant studies that met all criteria. Additional searches were conducted on epidemiology and treatment‐related biomarkers of bladder cancer to supplement the evidence. Among 41 studies, 24 reported on platinum‐based chemotherapy, eight on non‐platinum‐based chemotherapy, six on immunotherapy, two on targeted therapy, and one on surgery. Efficacy outcomes were summarized by line of therapy. Treatment‐related biomarkers including PD‐L1, HER2, and FGFR3 alterations were identified, and the alteration rate of FGFR3 of Chinese UC patients was lower than that of the western patients.

**Conclusions:**

Despite chemotherapy has been the main treatment choice for decades, appealing new therapeutic strategies including ICIs, targeted therapies and ADCs were applied in clinical practice. Further research on epidemiology and treatment‐related biomarkers of la/mUC patients is needed given only a limited number of studies have been identified thus far. High genomic heterogeneity and complexity of molecular features were observed among la/mUC patients; thus, further studies are required to identify critical drivers and promote potential precise therapies.

## 1 INTRODUCTION

Urothelial carcinoma (UC) characterizes a series of neoplasms derived from the urothelial endothelium, including the renal pelvis, ureter, bladder, and urethra.^1^ Among all the pathological types, UC is the predominant histologic type of bladder cancer (BC), which accounts for approximately 90% of BC.^1^ BC is the 10th and 13th most common cancer worldwide and in China in terms of incidence rate, with an estimated new cases of 573,278 globally and 85,694 in China in 2020.^2^ Locally advanced and metastatic urothelial carcinoma (la/mUC) was diagnosed in 7% and 5% of all patients with UC, respectively.^3^ The five‐year survival rates are only 34% for locally advanced UC and 5.4% for metastatic UC.^3^

According to the Chinese Urological Association (CUA) Guideline, the first‐line treatment for la/mUC who are eligible for platinum‐based chemotherapy is the combination therapy of cisplatin/carboplatin and gemcitabine.^4^ The initial responses to first‐line cisplatin‐based treatment could occur in up to 50%–70% of patients. However, the responses are rarely sustainable, with a median progression‐free survival (PFS) of 7 to 9 months and a median overall survival (OS) of 11–15 months.^5^, ^6^ Besides, those patients will eventually progress, leading to a median OS of only 6–9 months with poor prognosis.^6^

In recent years, several immune checkpoint inhibitors (ICIs) have been approved for patients who are not eligible for or having progressed through chemotherapy.^7^, ^8^ In China, tislelizumab was approved in 2020 for treating la/mUC patients with high PD‐L1 expression who fail platinum‐based chemotherapy, and toripalimab obtained the approval in 2021 for patients who fail platinum‐based chemotherapy.^8^ There remain uncertainty in efficacy and safety of ICIs, the objective response rates ranged from 24% to 30% for first‐line patients,^9^, ^10^ and patients experienced immune‐related adverse events (irAEs).^9^, ^10^ Given the perceived unmet needs of current treatments, there are continuing investigations in more novel therapies.^11^, ^12^, ^13^

Genomic profiling of UC has revealed common potentially actionable genomic alterations, indicating potential target therapies with driver molecular alterations, including fibroblast growth factor receptor (FGFR), human epidermal growth factor receptor 2 (HER2), and tumor mutational burden (TMB).^11^, ^14^, ^15^ In 2019, the Food and Drug Administration (FDA) approved erdafitinib, which is the first targeted agent for second‐line treatment of la/mUC patients with susceptible FGFR2 or FGFR3 genetic alterations.^12^ In addition, a series of antibody‐conjugated drugs (ADCs) have been developed, such as enfortumab vedotin (EV) targeting nectin‐4, RC48‐ADC targeting HER2, and sacituzumab govitecan targeting TROP‐2, which have been demonstrated promising activity in clinical trials.^16^

Based on our current knowledge, rare systematic review or scoping review summarized available evidence specifically for la/mUC patients in China, and there are only a few published systematic reviews for la/mUC patients focusing on population from outside China.^17^, ^18^, ^19^ A recent systematic review in la/mUC only reported the global epidemiology data of BC and introduced cisplatin/non‐cisplatin‐based regimens and ICIs in several developed countries.^17^ At the same time, there have been previous discussions on treatment‐related biomarkers that might be potential therapeutic targets; however, rare studies summarized the current evidence.^11^, ^12^ Epidemiology data of bladder cancer in China were reported in several studies, but few investigated the epidemiology of la/mUC patients.^20^, ^21^, ^22^ Therefore, the aim of this scoping review is to synthesize current evidence on epidemiology of la/mUC, such as prevalence, incidence and mortality; identify published articles to summarize current treatment options being studied and the corresponding efficacy and safety profiles; and investigate the treatment‐related biomarkers, including the detection technology, genomic alterations, and associated alteration rates and types among Chinese population. The results will be used to identify existing knowledge gap for la/mUC patients in China and provide potential research directions.

## 2 METHODS

To explore the widespread and heterogeneous evidence in topics of interest for la/mUC, a scoping review was conducted to summarize current knowledge and identify potential evidence gap. This study was conducted with the rigid and comprehensive procedure of scoping review, in accordance with the guideline developed by Joanna Briggs Institute (JBI).^23^ The processes of identification, screening, and inclusion of the study followed the recommendation of Preferred Reporting Items for Systematic Reviews and Meta‐Analyses extension for Scoping Reviews (PRISMA‐ScR).^24^

### 2.1 Search strategy

Search terms were developed with the guidance of Population, Concept, and Context framework shown in Supplement File, and preliminary searches were conducted to refine the full strategies (Supplement File). Free text words including “urinary bladder,” “urinary,” “urothelial,” “urothelial cell,” “transitional cell,” “bladder,” “urogenital tract,” “upper tract urothelial,” “ureteral,” “urologic,” “renal pelvis,” “urethra*,” “urothelium,” “tumor*,” “tumour*,” “neoplasm*,” “cancer,” “carcinoma*,” “stage 3,” “stage iii,” “stage 3a,” “stage iiia,” “stage 3b,” “stage iiib,” “stage 4,” “stage iv,” “metasta*,” “advanc*,” “relap*,” “recurrence*,” “refract*,” “late‐stage*,” OR “late stage*,” “inoperable,” “locally advanced,” “locally‐advanced” were used. Searches were limited to paper from peer‐reviewed journals published between January 1, 2011, and March 18, 2022, in both English and Chinese bibliographic databases, including PubMed, Embase, Web of Science, China National Knowledge Infrastructure (CNKI), and WANFANG.

### 2.2 Inclusion and exclusion criteria

Studies were included if they: (1) focused on Chinese patients (Mainland China, Taiwan, Hong Kong, Macau) with locally advanced or metastatic urothelial carcinoma, referring to histological or cytological stage in T3/4, or metastatic to pelvic lymph nodes (N+) or to lymph nodes, organs, or bones (M+); (2) were primary studies; (3) reported information related to epidemiology, existing treatment options and associated efficacy and safety, and genomic alterations and testing method of treatment‐related biomarkers based on large cohort studies; (4) were published in peer‐reviewed English journals, and Chinese journals from the list of Peking University Core Journals of China; and (5) were published between January 1, 2011, and March 18, 2022.

Studies were excluded if they: (1) included patients with other disease or early stage urothelial carcinoma; or if no specific result reported for la/mUC patients, more than 20% patients were adenocarcinoma, squamous cell carcinoma, and small cell carcinoma; (2) were case studies, review articles, meta‐analysis, protocols, posters, personal opinions, conference abstracts, or laboratory research (both in vivo and in vitro); and (3) were published in peer‐reviewed journals in language other than English and Chinese.

### 2.3 Study selection and data extraction

All search results were compiled to the citation management tool (EndNote), and duplications were removed. Two levels of screening were conducted by two independent reviewers (C.Y. and C.C.), and any disagreements were resolved by consensus or by discussion of a third senior reviewer (W.H.). After initial screening, author Q.X. developed a standardized data extraction form, including authors, years, article type, baseline characteristics, outcome, and results of all included studies (Supplement File). Data extractions were conducted by two independent reviewers (T.L. and C.Y.) and conflicts were resolved by a third senior reviewer (J.H.), while no attempt was made to assess the risk of bias for individual studies.

## 3 RESULTS

### 3.1 Search results

**FIGURE 1:**
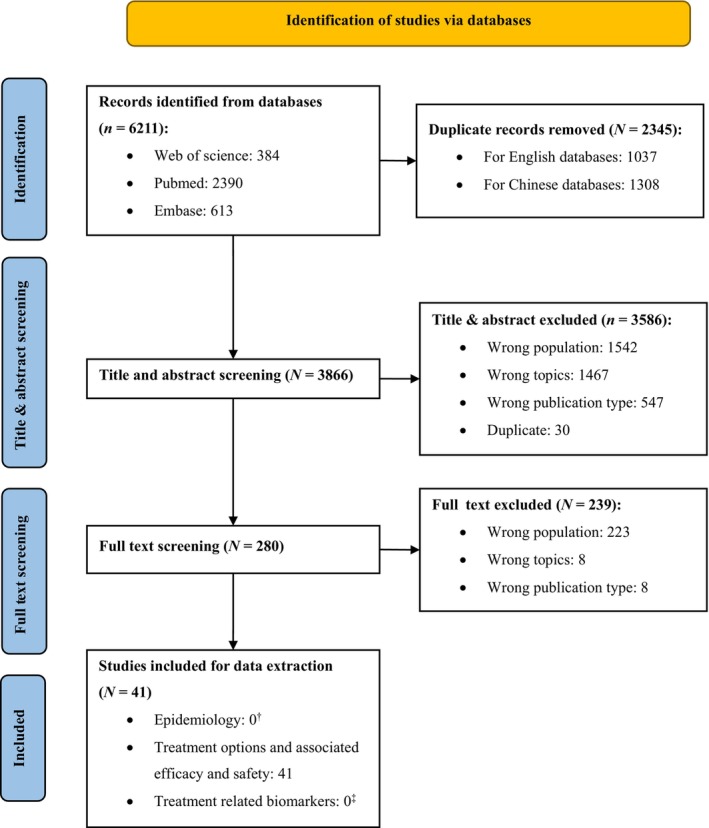
PRISMA flow chart of study selection. ^†^For epidemiological study, additional searches of national and international epidemiology websites as well as databases were conducted to identify the most recent epidemiology data for population with BC. ^‡^For treatment‐related biomarkers, studies were further identified as eligible if any biomarker testing results were reported. Besides, searches of FGFR alterations in UC patients were conducted with a total of 117 publications were identified in the selected databases and seven eligible studies were enrolled.

Among 6211 references screened, 41 unique studies were included after literature screening (Figure 1), with geographic representation of 20 regions in China. Of these studies, there were 35 single‐center and six multicenter studies, including 14 randomized controlled trials (RCTs), four single‐arm trials, nine prospective studies, and 14 retrospective studies.

The sample size of included studies ranged from 10 to 575, with proportion of males from 51% to 100%. Among 35 studies reported mean or median age, few studies included patients with a relatively young (*n* = 2, <50) or too old mean or median age (*n* = 3, >80), and the mean or median ages in most studies were reported between 50 and 59 year (*n* = 8) and between 60 and 69 years (*n* = 22). All patients included were la/mUC patients at stage III/IV. Of 26 studies included population with metastasis, median rates of metastasis in visceral, lymph node, and bone were 53%, 54%, and 17%, respectively.

Treatment options and associated efficacy and safety were investigated in all included studies, while no studies on epidemiology or treatment‐related biomarkers based on large cohort of Chinese la/mUC patients were retained. Noticing this literature gap, additional searches of international epidemiology websites, large China cancer registries, and literature databases were conducted to identify the most recent epidemiology data for BC. Since no articles on treatment‐related biomarkers were retained, further investigation loosed the inclusion criteria from large cohort studies to studies reporting any biomarker testing results. Among 41 included studies, biomarkers including PD‐L1 status and HER2 were mentioned in five studies. There were still no studies on FGFR; however, FGFR3 is demonstrated as the only validated predictive molecular marker in the 2021 update of the European Association of Urology Guidelines (EAU)^25^ and is recommended as the molecular testing for IIIB to IVB UC patients by the National Comprehensive Cancer Network (NCCN) guidelines.^1^ In addition, the Chinese Society of Clinical Oncology (CSCO), EAU, and NCCN guidelines suggest FGFR inhibitor as the second or subsequent line of treatment, and CUA guidelines encourages FGFR inhibitor as the treatment within clinical trials for la/mUC patients with FGFR2/3 alterations by biomarker testing.^1^, ^4^, ^25^, ^26^ Given this circumstance, supplementary searches were conducted to study FGFR in UC patients (Supplement File**)**, and seven eligible studies were included.

### 3.2 Incidence, prevalence, and mortality

According to the Global Cancer Statistics, the prevalence of BC in Mainland China was 16.3 per 100,000 population in 2020.^2^ National Central Cancer Registry and Global Cancer Statistics revealed that the crude incidence rate slightly increased from 5.7 per 100,000 to 5.9 per 100,000 from 2014 to 2020, and the age‐standardized incidence rate by world standard population (ASIRW) remained relatively stable at 3.6 per 100,000 in Mainland China. Given the same period, the crude mortality rate increased from 2.4 per 100,000 to 2.7 per 100,000 and the age‐standardized mortality rate by world standard population (ASMRW) increased from 1.3 per 100,000 to 1.6 per 100,000. In general, the incidence and mortality rates of males were higher than females. The incidence and mortality rates of urban areas were higher than those of rural areas in mainland China.^2^, ^20^, ^21^, ^22^, ^27^ The incidence and mortality of BC patients in Taiwan were extracted from Health Promotion Administration (HPA),^28^ which showed that from 2014 to 2019, ASIRW of males decreased from 8.0 per 100,000 to 7.3 per 100,000 and ASIRW of females decreased from 2.9 per 100,000 to 2.6 per 100,000. In addition, the proportion of stage III or IV BC patients at primary diagnosis decreased from 15.9% in 2017 to 12.5% in 2019 in Taiwan, with males accounting for twice the proportion of females.^28^

### 3.3 Efficacy and safety of available treatment options

Among 41 studies, five types of treatment regimens were identified, including platinum‐based and non‐platinum‐based chemotherapy, immunotherapy, targeted therapy and surgery, categorized based on the CSCO guideline.^26^ Twenty‐four studies investigated platinum‐based regimens, including eight RCTs, eight prospective studies, and eight retrospective studies. Of these, 23 studies were single‐center studies, while one RCT did not specify the number of sites.^29^ Details of study designs of all treatments are summarized in Table 1.

**TABLE 1:** Summary of study designs per treatment category.

Treatment category	RCT (*n*)	Single‐arm trial (*n*)	Prospective study (*n*)	Retrospective study (*n*)
Platinum‐based chemotherapy	8	0	8	8
Non‐platinum‐based chemotherapy^a^	6	0	1	1
Immunotherapy^b^	0	2	0	4
Targeted therapy^c^	0	1	1	0
Surgery^d^	0	0	0	1

^a^ Eight studies on non‐platinum‐based chemotherapy were all conducted in single center.

^b^ Two single‐arm trials were multicenter studies while the four retrospective studies were all conducted in single center.

^c^ For targeted therapy, one multicenter, prospective study on RC48‐ADC and one single‐center, single‐arm trial on PARP inhibitor were included.

^d^ The study of surgery was a single‐center study investigating percutaneous cryotherapy.

#### 3.3.1 Platinum‐based chemotherapy

Among 24 studies on platinum‐based regimens, 11 studies investigated patients receiving first line of therapy (LOT), two investigated second‐line therapy, and four investigated mixed line of therapy, while seven studies did not report LOT details (Table 2). Most studies investigated cisplatin or gemcitabine plus cisplatin (GC) therapy with or without other agents, including commonly used pirarubicin,^30^ docetaxel,^31^ or paclitaxel.^32^ For all platinum‐based studies, reported PFS ranged from 3.6 to 11 months, whereas OS ranged from 7.2 to 18 months.^33^, ^34^ The shortest PFS and OS were reported in one study comparing cisplatin‐based chemotherapy versus carboplatin‐based chemotherapy in elderly patients older than 70 years old.^33^

**TABLE 2:** Studies reporting platinum‐based chemotherapy.

Author year	Study design	Region	Study period	Population	Sample size	Patients by LOT, *n*	Therapy investigated	Control	Outcome
Wang HB, 2011^29^	Multicenter, RCT	Tangshan, Hebei	2006–2008	Advanced BC III‐IV (cT3‐cT4)	37	N/A	GC + hydroxycamptothecin + radiotherapy	GC + radiotherapy	CR 21.05% vs. 16.67%; ORR 84.21% vs. 50% (*p* < 0.05); two‐year survival 13.33% vs. 12.83% (*p* > 0.05)
Li FL, 2013^30^	Single center, RCT	Huizhou, Guangdong	2010–2012	Advanced BC III‐IV (cT3‐cT4)	70	N/A	GC + pirarubicin	GC + mitomycin	CR 14.3% vs. 8.6% (*p* = 0.452); ORR 65.7% vs. 31.4% (*p* = 0.004)
Luo ST, 2015^50^	Single center, RCT	Luzhou, Sichuan	2011–2013	mUC III‐IV (cT3‐cT4)	47	N/A	GC + pirarubicin	GC	CR 34.8% vs. 4.2%; ORR 78.3% vs. 41.7% (*p* < 0.05)
Liang XB, 2017^49^	Single center, RCT	Zhengzhou, Henan	2012–2015	Advanced urinary urothelial carcinoma, IV	78	N/A	Pemetrexed + cisplatin	Paclitaxel + cisplatin	CR 0% vs. 0%; ORR 41.03% vs. 43.59% (*p* > 0.05); one‐year survival 38.46% vs. 35.9% (*p* > 0.05)
Xiong JH, 2018^39^	Single center, RCT	Jiangxi	2013–2015	Advanced BC III‐IV (cT3‐cT4)	76	1L: 76	GC + radiotherapy	GC	CR 23.68% vs. 13.16% (*p* < 0.05); ORR 76.32% vs. 50% (*p* < 0.05) two‐year survival 21.05% vs. 13.16% (*p* > 0.05)
Zheng W, 2018^40^	Single center, RCT	Hangzhou, Zhejiang	2012–2015	mUC III‐IV	60	1L: 60	Docetaxel + cisplatin + Vascular endothelial growth inhibitor	Docetaxel + cisplatin	CR 16.67% vs. 10%; ORR 76.67% vs. 53.33% (*p* < 0.05)
Huang YQ, 2019^42^	Single center, RCT	Chengdu, Sichuan	2014–2016	Advanced BC III‐IV	575	N/A	GC + pirarubicin	GC	CR 29.07% vs. 9.79%; ORR 77.51% vs. 51.75% (*p* < 0.05); two‐year survival 61.25% vs. 35.31% (*p* < 0.05)
Wu JQ, 2019^38^	Single center, RCT	Xuzhou, Jiangsu	2012–2015	mUC III‐IV (T3‐4)	70	1L: 35	Pemetrexed + cisplatin	GC	CR 0% vs. 0% (*p* > 0.05); ORR 65.7% vs. 62.9% (*p* > 0.05) mPFS 6.5 vs. 6.9 months (*p* > 0.05); mOS 16 vs. 14 months (*p* > 0.05)
Chen Y, 2011^34^	Single center, prospective study	Shanghai	2004–2009	Advanced BC IV	33	1L: 33	GC	N/A	CR 27.27%; ORR 48.5%; mPFS 11 months; mOS 18 months
Ding B, 2011^45^	Single center, prospective study	Jinan, Shandong	2009–2011	mUC	52	1L: 17; ≥2L: 35	GC	N/A	CR 15.3%; ORR 63.5%
Zhang ZY, 2011^48^	Single center, prospective study	Luoyang, Henan	2007–2010	Advanced BC	56	1L: 25; neoadjuvant after surgery: 31	Gemcitabine + oxaliplatin	N/A	CR 5.36%; ORR 43.57%
Chen R, 2013^41^	Single center, prospective study	Urumqi, Xinjiang	2006–2011	mUC III‐IV (cT4)	30	1L: 30	GC	N/A	CR 10%; ORR 60%
Xie F, 2014^52^	Single center, prospective study	Jinan, Shandong	2009–2012	mUC III‐IV (cT3b‐cT4)	83	N/A	Lifein + GC	GC	CR 15.4% vs. 11.9%; ORR 66.7% vs. 50% (*p* = 0.18)
Ren Q, 2014^37^	Single center, prospective study	Guilin, Guangxi	2008–2010	Advanced BC	26	1L: 26	GC	N/A	CR 19.23%; ORR 46.2%; mPFS 10.8 months; mOS 17.6 months
Xing XH, 2014^47^	Single center, prospective study	Hainan	2007–2012	Advanced BC III‐IV	48	1L: 48	Docetaxel + cisplatin	N/A	CR 12.5%; ORR 64.58%; mPFS 10.8 months; mOS 16.7 months
He JP, 2015^32^	Single center, prospective study	Chengdu, Sichuan	2014–2014	mUC	10	1L: 10	GC + paclitaxel	N/A	CR 10%; ORR 50%
Hu LB, 2012^51^	Single center, retrospective study	Kunming, Yunnan	2006–2008	mUC	52	N/A	GC	N/A	CR 7.69%; ORR 48.08%
Hsieh M, 2015^35^	Single center, retrospective study	Taiwan	1997–2014	mUC	203	1L: 147; 2L: 53	MVAC	GC	UTUC patients: CR 31% vs. 3%; ORR 61% vs. 46% (*p* = 0.1); mPFS 7.3 vs. 4.0 months (*p <* 0.001); mOS 17.0 vs. 10.5 months (*p <* 0.001) UC patients: CR 29% vs. 13%; ORR 64% vs. 60% (*p* = 0.7); mPFS 6.8 vs. 6.3 months (*p* = 0.35); mOS 16.3 vs. 13.0 months (*p* = 0.06)
Xie J, 2016^31^	Single center, retrospective study	Beijing	2013–2014	mUC	50	N/A	Docetaxel + cisplatin	N/A	mOS 15.4 months
Zhang S, 2016^44^	Single center, retrospective study	Shanghai	2008–2011	la/mUC	33	2L: 33	Oxaliplatin + 5‐fluorouracil (5‐FU) + leucovorin (LV) (FOLFOX)	N/A	CR 0%; ORR 27%; mean PFS 3 months; mean OS 6.1 months
Zang L, 2017^43^	Single center, retrospective study	Tianjin	2015–2017	la/mUC IV	11	2L: 11	Pemetrexed + nedaplatin	N/A	CR 18.18%; ORR 63.6%; mPFS 7 months
Hsieh M, 2018^36^	Single center, retrospective study	Taiwan	1997–2015	mUC	141	1L: 141	Carboplatin‐based chemotherapy; Cisplatin‐based chemotherapy; other chemotherapy	N/A	mOS 13.5 months
Liaw CC, 2019^46^	Single center, retrospective study	Taiwan	2008–2017	mUC	139	Most patients received 1L therapy while a few received 2L therapy	5‐Aconitase + folinic acid calcium salt pentahydrate + GC	N/A	ORR 60%; mOS 17 months; five‐year survival: 18%
Huang SY, 2020^33^	Single center, retrospective study	Taiwan	2001–2018	mUC	108	1L: 108	Cisplatin‐based chemotherapy	Carboplatin‐based chemotherapy	CR 4.4% vs. 2.5% (*p* = 0.61); ORR 41.2% vs. 32.5% (*p* = 0.42); mPFS 5.2 vs. 3.6 months (*p* = 0.19); mOS 13.6 vs. 7.2 months (*p* = 0.045)

Abbreviations: 1L, first line; 2L, second line; CR, complete response; GC, gemcitabine + cisplatin; la/mUC, locally advanced/metastatic urothelial carcinoma; LOT, line of therapy; mOS, median overall survival; mPFS, median progression‐free survival; MVAC, methotrexate + vinblastine + doxorubicin + cisplatin; N/A, not applicable; ORR, objective response rate; UC, urothelial carcinoma; UTUC, upper urinary tract urothelial carcinoma.

Within 11 studies investigating first‐line therapies,^32^, ^33^, ^34^, ^35^, ^36^, ^37^, ^38^, ^39^, ^40^, ^41^, ^42^ five studies were single‐arm design,^32^, ^34^, ^36^, ^37^, ^41^ while six studies compared different regimens.^33^, ^35^, ^38^, ^39^, ^40^, ^42^ Efficacy and safety outcomes varied among different platinum‐based regimens. The complete response (CR) of first‐line standard chemotherapy, gemcitabine plus cisplatin (GC) therapy, ranged from 0 to 27.27%,^34^, ^38^ while the objective response rate (ORR) ranged from 46% to 62.9%.^35^, ^38^ PFS of first‐line GC therapy ranged from 4 to 11 months while OS ranged from 10.5 to 18 months.^34^, ^35^ Overall survival reported by other first‐line platinum‐based chemotherapies such as methotrexate, vinblastine, doxorubicin, and cisplatin (MVAC),^35^ or pemetrexed plus cisplatin therapy^38^ was within the range of 10.5 to 18 months.

Two studies investigated nedaplatin and oxaliplatin on patients who had failed prior cisplatin‐based chemotherapy, which demonstrated nedaplatin and oxaliplatin as well‐tolerated second‐line regimens.^43^, ^44^ One single‐center retrospective study evaluating oxaliplatin plus 5‐fluorouracil (5‐FU) and leucovorin (LV) (FOLFOX) reported an ORR of 27% and a median OS of 6.1 months.^44^ Eleven patients receiving nedaplatin plus pemetrexed as second‐line treatment after failure of platinum‐based chemotherapy reported an ORR of 63.6% and a median PFS of 7 months.^43^ Two studies investigated platinum‐based regimens on treatment‐naive or treatment‐experienced patients,^45^, ^46^ with ORR ranging from 60% to 63.5%,^45^, ^46^ and OS of 17 months were reported in one study.^46^ Two studies investigated cisplatin‐based regimens on treatment‐naive patients and patients receiving chemotherapy after surgery, reporting ORR of 43.57% to 64.58% and 16.7 months of median OS.^47^, ^48^

Seven studies did not report information on LOT,^29^, ^30^, ^31^, ^49^, ^50^, ^51^, ^52^ among which one study found that patients with only lymph node metastasis had better ORR compared to patients with visceral metastasis (64.29% vs. 29.17%, *p* < 0.05).^51^ Furthermore, Xie et al conducted univariate survival analysis on patients with mUC undergoing chemotherapy and found that bone metastasis (*p* = 0.005), visceral metastasis (*p* = 0.032), multiple metastasis (*p* = 0.024), and number of visceral metastasis ≥3 (*p* = 0.032) were associated with the poor prognosis of mUC patients.^31^ Most frequent reported AEs of grade ≥3 and the incidence rate ≥ 10% from included studies on platinum‐based chemotherapy were leukopenia, thrombocytopenia, nausea or vomiting, neutropenia, and anemia.^34^, ^41^, ^43^, ^48^

#### 3.3.2 Non‐platinum‐based chemotherapy

A total of eight studies were identified to evaluate the efficacy and safety of non‐platinum‐based chemotherapy, as shown in Table 3. One study reported that gemcitabine and epirubicin (GE) chemotherapy was chosen because it was more tolerable for elderly patients compared to GC chemotherapy^53^ while the other seven studies did not illustrate whether patients were cisplatin ineligible or not. First‐line chemotherapy was investigated in three studies, with one of gemcitabine plus docetaxel (GD) compared to GC,^54^ one of GD combined with three‐dimensional conformal radiotherapy (3D‐CRT) compared to GD,^55^ one of gemcitabine plus paclitaxel (GT) compared to gemcitabine plus oxaliplatin.^56^ One study evaluated capecitabine and docetaxel versus capecitabine in treatment‐naive and experienced patients.^57^ The remaining four studies did not report LOT.^53^, ^58^, ^59^, ^60^ Among the four studies, one study reported extraordinarily high ORR of 95.7% of 5‐fluorouracil (5‐Fu) plus hydroxycamptothecin (HCPT) compared to 76.1% of 5‐Fu (*p* = 0.007).^60^

**TABLE 3:** Studies reporting non‐platinum‐based chemotherapy.

Author year	Study design	Region	Study period	Population	Sample size	Patients by LOT, *n*	Therapy investigated	Control	Outcome
Zhang JK, 2011^54^	Single center, RCT	Zhongshan, Guangdong	2004–2007	Advanced UC III‐IV (cT3‐cT4)	43	1L	GD	GC	CR 4.8% vs. 0% (*p* > 0.05); ORR 38.1% vs. 31.6% (*p* > 0.05); mean PFS 9 vs. 10 months (*p* > 0.05); mean OS 16.5 vs. 15.7 months (*p* > 0.05)
Huang JP, 2014^56^	Single center, RCT	Qianjiang, Hubei	2009–2011	Advanced BC III‐IV	92	1L	GT	Gemcitabine + oxaliplatin	CR 25.5% vs. 17.1% (*p* = 0.039); ORR 58.8% vs. 53.7% (*p* = 0.036); mOS 17.1 vs. 12.8 months (*p* < 0.05)
Su HY, 2015^58^	Single center, RCT	Shenyang, Liaoning	2009.1–2009.12	Advanced BC III‐IV	80	N/A	Pirarubicin + docetaxel	Pirarubicin	CR 50% vs. 27.5%; ORR 82.5% vs. 52.5% (*p* < 0.05) five‐year survival 52.5% vs. 37.5% (*p* < 0.05)
Wang JW, 2015^59^	Single center, RCT	Baoji, Shanxi	2010–2012	Advanced BC II–IV (T2–T4)	93	N/A	GT + 3D‐CRT	GT + regular radiotherapy	CR 40.8% vs. 18.2% (*p* < 0.05); ORR 61.2% vs. 52.3% (*p* < 0.05); mOS 17.3 vs. 11.6 months (*p* < 0.05)
Luo LP, 2016^60^	Single center, RCT	Wuhan, Hubei	2011–2015	Advanced BC III‐IV (cT3‐cT4)	92	N/A	5‐Fu + HCPT	5‐Fu	CR 65.22% vs. 28.26%; ORR 95.7% vs. 76.1% (*p* = 0.007)
Chen LJ, 2018^55^	Single center, RCT	Xinxiang, Henan	2015–2016	Advanced BC III‐IV	96	1L: 96	GD + 3D‐CRT	GD	CR 20.83% vs. 16.67% (*p* < 0.05); ORR 79.17% vs. 58.33% (*p* < 0.05); two‐year survival 43.75% vs. 22.92% (*p* = 0.023)
Xia YL, 2011^53^	Single center, prospective study	Tangshan, Hebei	2004–2008	Advanced BC	62	N/A	GE+3D‐CRT	GE	CR 18.8% vs. 10%; ORR 75% vs. 46.7% (*p* < 0.05); mOS 21 vs. 14 months
Xue C, 2016^57^	Single center, retrospective study	Guangzhou, Guangdong	2009–2015	la/mUC	29	1 L: 8 2 L: 21	capecitabine and docetaxel	capecitabine	PR 18.2% vs. 0%; mPFS 2.2 vs. 3 months (*p* = 0.81); mOS 18 vs. 11.3 months (*p* = 0.771)

Abbreviations: 1L, first line; 2L, second line; 3D‐CRT, three‐dimensional conformal radiotherapy; 5‐Fu, 5‐fluorouracil; BC, bladder cancer; CR, complete response; GC, gemcitabine/cisplatin; GD, gemcitabine/docetaxel; GE, gemcitabine/epirubicin; GT, gemcitabine/paclitaxel; HCPT, hydroxycamptothecin; la/mUC, locally advanced/metastatic urothelial carcinoma; LOT, line of therapy; mOS, median overall survival; mPFS, median progression‐free survival; N/A, not applicable; ORR, objective response rate; PR, partial response; UC, urothelial carcinoma.

Three studies evaluated 3D‐CRT combined with chemotherapy compared to chemotherapy with or without two‐dimensional conventional radiotherapy, and all three studies observed a significant difference in ORR between the observation group and control group.^53^, ^55^, ^59^ One study reported a significant difference in OS (17.3 vs. 11.6 months, *p* < 0.05) comparing 3D‐CRT combined with GT chemotherapy to regular radiotherapy combined with GT chemotherapy,^59^ whereas median OS was not reported in other two studies.^53^, ^55^ In terms of AEs, grade ≥3 AEs of neutropenia, leukopenia, thrombocytopenia, and anemia were reported in two studies with occurrence rates ≥10%.^54^, ^57^ The other six studies only observed AEs in grade 1–2 or the occurrence rate of grade 3 or above AE was less than 10%.

#### 3.3.3 Immunotherapy

Immunotherapies were reported in six studies, two of which were single‐arm trials and four of which were retrospective cohort studies.^15^, ^61^, ^62^, ^63^, ^64^, ^65^ Study details are provided in Table 4. Treatment‐specific data were reported in single‐arm trials for tislelizumab and toripalimab respectively, while the remaining four studies reported data for multiple immunotherapies.^15^, ^61^ Han et al retrospectively reported anti‐PD‐1 agents combined with chemotherapy,^63^ compared with chemotherapy as first‐line treatment. Han et al. demonstrated that patients in the immunotherapy plus chemotherapy group had significantly better CR and PFS than the chemotherapy group, while adverse events were not significantly different. OS of the immunotherapy plus chemotherapy group was not reached, while OS of the chemotherapy group was 17.8 months.^63^

**TABLE 4:** Studies reporting immunotherapy.

Author year	Study design	Region^a^	Study period	Population	Sample size	Patients by LOT, n	Therapy investigated	Control	Outcome	PD1/L1 expression status, n (%)	PD1/L1 testing method and positive definition
Ye D, 2021^61^	Multicenter, single‐arm trial	Mainland China	2019.09	la/mUC	113	1L: 21 2L: 73 3L: 19	Tislelizumab monotherapy	N/A	CR 10%; ORR 24%; mPFS 2.1 months; mOS 9.8 months	TC <50% and IC <50%, 77 (68) TC ≥50% or IC ≥50%, 36 (32)	IHC
Sheng X, 2022^15^	Multicenter, single‐arm trial	Mainland China	2017–2019	la/mUC IIIb‐IV	151	1L: 14 2L: 105 ≥3L: 32	Toripalimab monotherapy	N/A	CR 1%; ORR 26%; mPFS 2.3 months; mOS 14.4 months patients with primary tumor sites in the upper urinary tracts, ORR 27%; patients with primary tumor sites in the lower urinary tracts, ORR 24%	Positive, 48 (32) Negative, 96 (64) Unknown, 7 (5)	IHC. PD‐L1 positive was defined as the presence of membrane staining of any intensity in ≥1% of TC
Kuo M, 2020^62^	Multicenter, retrospective study	Taiwan	2016–2019	mUC	129	1L: 97; 2L: 19; 3L: 13 (ESRD:11; Non‐ESRD: 118)	PD‐1/PD‐L1 monotherapy; PD‐1/PD‐L1 + Chemotherapy; PD‐1/PD‐L1 + Anti‐CTLA‐4 Anti‐PD‐1: Nivolumab, Pembrolizumab Anti‐PD‐L1; Atezolizumab, Durvalumab, Avelumab	N/A	mUC with ERSD vs. mUC without ERSD CR 0% VS. 12.7%; ORR 54.5% VS. 28.8% (*p* = 0.09); mPFS 7.1 vs. 3.5 months (*p* = 0.42); mOS not reached vs. 15.4 months	71 patients tested PD‐L1: TPS≧1, 37 (52.1) TPS≧10, 27 (38)	IHC
Han FX, 2021^63^	Single center, retrospective study	Beijing	2017–2020	mUC IIIb‐IV	81	1L: 81	PD‐1 (Pembrolizumab, Nivolumab, Sintilimab) + chemotherapy	Gemcitabine/paclitaxel+Platinum therapy: 51	CR 13.33% vs. 1.96% (*p* = 0.049); ORR 46.67% vs. 49.02% (*p* = 0.999) mPFS 15.8 vs. 7.17 months (*p* = 0.02); mOS not reached vs. 17.8 months	N/A	N/A
Hsieh S, 2021^64^	Multicenter, retrospective study	Taiwan	2019–2020	la/mUC	145	1L: 33 2L: 112	Nivolumab/pembrolizumab/atezolizumab monotherapy	N/A	1 L: CR 12.12%; ORR 42.4%; mPFS 2.7 months 2 L: CR 9.82%; ORR 40.2%; mPFS 3.8 months	N/A	N/A
Li H, 2021^65^	Single center, retrospective study	Guangzhou	2015–2019	mUC	52	1L: 24 2L: 28	PD‐1/PD‐L1‐based treatment	N/A	mPFS 6.2 months; mOS 20.53 months	Positive, 4 (7.69) Negative, 12 (23.08) Unknown, 36 (69.23)	IHC. PD‐L1 positive was defined as the presence of membrane staining of any intensity in >1% of TC

Abbreviations: 1L: first line; 2L, second line; Anti‐CTLA‐4, anti‐cytotoxic T‐lymphocyte‐associated protein (CTLA)‐4; CR, complete response; ESRD, end‐stage renal disease; IHC, immunohistochemistry; la/mUC, locally advanced/metastatic urothelial carcinoma; LOT, line of therapy; mOS, median overall survival; mPFS, median progression‐free survival; N/A, not applicable; ORR, objective response rate; PD‐1, programmed cell death protein 1; PD‐L1, programmed cell death ligand 1; TC, tumor cell; TPS, tumor proportion score.

^a^ Mainland China indicated that no specific information about region was reported.

Two single‐arm studies investigating tislelizumab and toripalimab demonstrated different efficacy profiles between PD‐L1+ and PD‐L1‐ subgroups.^15^, ^61^ In the first study, 113 patients with PD‐L1+ UC who progressed during or following a platinum‐based regimen were administered with tislelizumab.^61^ CR was reported as 10% while ORR was 24%. Furthermore, ORR was analyzed by different population groups. Higher ORRs were observed in the subgroup of metastasis of lymph nodes only. ORRs in the subgroup of higher PD‐L1 expression (TC ≥50% or IC ≥50%) were observed to be numerically higher than those in the subgroup of lower PD‐L1 expression (TC <50% and IC <50%) (29.4% vs. 21.4%, *p* value not reported). The other study investigating toripalimab enrolled 151 patients with varying PD‐L1 status,^15^ and PD‐L1‐positive patients defined by TC positive staining ≥1% had significantly better ORR and PFS than PD‐L1‐negative patients (ORR, 42% vs. 17%, *p* = 0.002; median PFS, 3.7 vs. 1.8 months, *p* = 0.001). Median OS was not observed significant difference between PD‐L1+ patients and PD‐L1‐ patients (35.6 vs. 11.2 months, *p* = 0.49).

Within the three retrospective studies, one study retrieved data from the Taiwan registration system which reported outcomes of UC patients receiving first‐line (ORR, 42.2%; PFS, 2.7 months) and second‐line treatment (ORR 40.2%, PFS 3.8 months).^64^ One study with no specific PD1/L1 treatment reported median PFS as 6.2 months and OS as 20.53 months in 52 treatment‐naive and treatment‐experienced mUC patients.^65^ The other study found that immunotherapy might be beneficial for UC patients with ESRD.^62^

In terms of safety, treatment‐related adverse events (TRAE) and immune‐related adverse events (irAE) were reported. For grade ≥3 and incidence rates ≥5% TRAE, anemia, thrombocytopenia, neutropenia, leukopenia, skin rash, and hyponatremia were reported. Only two studies reported irAEs.^61^, ^64^ Patients receiving tislelizumab were observed to have grade 3–4 irAEs, including skin reaction (3%), hepatitis (3%), pancreatitis (2%), nephritis and renal dysfunction (0.88%), and colitis (0.88%), respectively.^61^ Real‐world data from Taiwan revealed that 11.5% and 8.8% of mUC patients receiving first‐line and second‐line PD1/L1 therapy reported irAEs, respectively.^64^

#### 3.3.4 Targeted therapy

Two studies (Table 5) investigated targeted therapy with one RC48‐ADC and one poly adenosine diphosphate‐ribose polymerase (PARP) inhibitor, olaparib.^11^, ^66^ In a multicenter single‐arm trial, RC48‐ADC was administered to previously treated patients (2L: 31; ≥3L:12) with HER2 expression positive detected by the immunohistochemistry (IHC) staining method.^11^ In this study, patients were evaluated by IHC as HER2 2+ or HER2 3+ (HER2 2+ and HER2 3+ were defined as HER2+). Gene amplification of these patients was evaluated by fluorescence in situ hybridization (FISH) with a 46.5% positive rate. For patients with HER2 IHC3+ or IHC2+ and FISH+, ORR was 60%, while ORR was 40% for patients with HER2 IHC2+ and FISH‐. The median PFS and OS for patients in the study were 6.9 and 13.9 months, respectively. For grade 3 TRAEs with an occurrence rate ≥10%, hypoesthesia (23.3%) and neutrophil count decrease (14%) were reported. Regarding another targeted therapy, olaparib was investigated in a multicenter single‐arm trial, showing that the OS rate of the DNA repair deficiency group was higher than the DNA repair normal group (eight‐week survival, 80% vs. 66%, *p* < 0.05).^66^ The incidence rates of AEs including neutropenia, thrombocytopenia, anemia, fatigue, and leukopenia in the two groups had no significant difference (*p* > 0.05).

**TABLE 5:** Studies reporting targeted therapy and surgery.

Author year	Study design	Region^a^	Study period	Population	Sample size	Patients by LOT, n	Therapy investigated	Control	Outcome
Zhu B, 2016^66^	Multicenter, single‐arm trial	Fushun	2015.2–2015.9	mUC	100	N/A	Olaparib (DNA repair deficiency group, DNA repair normal group)	N/A	Eight‐week survival: repair deficiency vs. repair normal 80% vs. 66% (*p* < 0.05)
Sheng X, 2021^11^	Multicenter, single‐arm trial	Mainland China	2017–2018	la/mUC	43	2L: 31; ≥3L: 12	RC48‐ADC	N/A	CR 0%; ORR 51.2%; mPFS 6.9 months; mOS 13.9 months
Zhou L, 2014^67^	Single center, retrospective study	Guangzhou	2007.10–2011.09	mUC	23	Mixed lines	Percutaneous cryotherapy	N/A	mPFS 17 months

Abbreviations: 2L, second line; 3L, third line; CR, complete response; la/mUC, locally advanced/metastatic urothelial carcinoma; LOT, line of therapy; mOS, median overall survival; mPFS, median progression‐free survival; N/A, not applicable; ORR, objective response rate;

^a^ Mainland China indicated that no specific information about region was reported.

#### 3.3.5 Surgery

A retrospective study investigated 23 patients receiving percutaneous cryotherapy who refused systemic chemotherapy or failed previous chemotherapy (see Table 5).^67^ PFS after cryosurgery was 17 months for 11 patients with UC in the metastatic stage of the disease among included patients. The study concluded that percutaneous cryotherapy may be a safe and efficacious treatment option for metastatic bladder cancer.

### 3.4 Treatment‐related biomarkers among Chinese population

Among 12 eligible studies reporting treatment‐related biomarkers, five reported patients with PD‐L1 and HER‐2 expressions and seven revealed the FGFR alterations in Chinese UC patients.

In four studies reporting PD‐L1 expression, positive rates of PD‐L1 ranged from 33.33% to 100% among those tested via IHC due to different enrollment criteria of patients dependent on studies.^15^, ^61^, ^62^, ^65^ One study only included PD‐L1+ patients^61^ whereas other three studies included PD‐L1 status‐negative or ‐unknown patients.^15^, ^62^, ^65^ In addition, one study investigated RC48‐ADC in HER2‐positive patients evaluated by IHC and further confirmed by FISH.^11^

In seven studies related to FGFR, five reported FGFR3 alterations,^68^, ^69^, ^70^, ^71^, ^72^ one explored FGFR family alterations in UC patients,^73^ and one investigated the landscape of FGF/FGFR alterations in a large cohort of 12,737 cancer patients with a high prevalence of FGF/FGFR mutations occurring in urinary tract cancer (52.7%, 145/275).^74^ Various testing methods were applied, including sanger sequencing (*n* = 2), next‐generation sequencing (NGS, *n* = 4), and whole‐genome sequencing (*n* = 1).^68^, ^69^, ^70^, ^71^, ^72^, ^73^, ^74^

FGFR3 mutations of UC originated in different tumor locations have been investigated, its mutations in UTUC and UC patients were reported in three studies, while no significant difference in proportions was identified.^68^, ^72^, ^73^ Two studies reported on western cohorts downloaded from cancer genome databases, however, revealed a significantly higher mutated frequency of FGFR3 in upper tract urothelial carcinoma (UTUC) compared to UC patients.^72^, ^73^ One study investigated a cohort consisting of 16 non‐muscle invasive bladder cancer (NMIBC) and 16 muscle invasive bladder cancer (MIBC) fresh samples from Chinese patients, showing FGFR3 mutation rates of 50% (8/16) in NMIBC and 12.5% (2/16) in MIBC, respectively.^71^

FGFR3 mutation rates were reported in six studies,^68^, ^69^, ^70^, ^71^, ^72^, ^73^ ranging from 2.61% to 31.2% (Table 6). Comparisons between different cohorts were conducted in three studies, indicating a lower mutation rate of Chinese cohorts compared to western cohorts.^68^, ^72^, ^73^ Furthermore, one of these studies reported that FGFR3 alteration in the western UTUC cohort was three times higher than that of the Chinese cohort (48.24% vs. 16.13%, *p* < 0.001).^73^ In general, unique genomic features of Chinese UC patients were found, comparing to western patients in these studies.

**TABLE 6:** Studies reporting genetic alterations.

Author year	Target expression	Gene expression	Target population	Testing method	Frequency among tested population	Alteration region
Zuo W, 2020^74^	FGF/FGFR	Mutation	Urinary tract cancer	NGS	52.7%	N/A
Yuan X, 2016^68^	FGFR3	Mutation	BC	Sanger sequencing	9.48%	A248C, S249C, G372C, L652G
	FGFR3	Mutation	Renal Pelvis Carcinoma	Sanger sequencing	8.79%	A248C, S249C, G327C, S373C, T375C
	FGFR3	Mutation	Ureter Carcinoma	Sanger sequencing	2.61%	A248C, S249C, L652G
Guo G, 2013^69^	FGFR3	Mutation	BC	Whole‐genome Sequencing	14.14%	N/A
Wang K, 2015^70^	FGFR3	Mutation	BC	Sanger sequencing	6.86%	R248C, S249C, G372C, Y375C
Wang T, 2020^71^	FGFR3	Mutation	BC	NGS	31.30%	N/A
Yang B, 2021^73^	FGFR1	Alteration (some were amplification)	UC	NGS	5%	N/A
	FGFR2	Mutation /amplification	UC	NGS	8%	G305A, T207P, M803L, G683T
	FGFR3	Alteration (some were fusion)	UC	NGS	13%	S249C, A248C, T373C, H349A, V166M, T755L
	FGFR4	Alteration	UC	NGS	6%	N/A
Yang K, 2021^72^	FGFR3	Mutation	BC	NGS	18.00%	N/A
	FGFR3	Mutation	UTUC	NGS	20.00%	N/A
Kuo M, 2020^62^	PD‐L1	Positive	mUC	IHC	52.10%	N/A
Li H, 2021^65^	PD‐L1	Positive	mUC	IHC	25%	N/A
Ye D, 2020^61^	PD‐L1	Positive	la/mUC	IHC	100%	N/A
Sheng X, 2022^15^	PD‐L1	Positive	mUC	IHC	33.33%	N/A
Sheng X, 2021^11^	HER2	Positive	la/mUC	IHC	100%	N/A
	HER2	Positive	la/mUC	FISH	46.50%	N/A

Abbreviations: BC, bladder carcinoma; FISH, fluorescence in situ hybridization; IHC, immunohistochemistry; la/mUC, locally advanced/metastatic urothelial carcinoma; mUC, metastatic urothelial carcinoma; N/A, not applicable; NGS, next‐generation sequencing; UC, urothelial carcinoma; UTUC, Upper tract urothelial cancer.

## 4 DISCUSSION

This scoping review aims to understand Chinese la/mUC patients covering the topics of epidemiology, effectiveness and efficacy regarding available treatments, and treatment‐related biomarkers. Through conducting comprehensive literature review and qualitative analysis of reviewed studies, we summarized up‐to‐date incidence, prevalence, and mortality rates of bladder cancer of China. In addition, by investigating the treatment landscape for la/mUC, including platinum‐based chemotherapy, non‐platinum‐based chemotherapy, immunotherapy, targeted therapy, and surgery, this review offered evidence‐based support to help clinicians with their treatment decision‐making. Furthermore, treatment‐related biomarkers specified for Chinese patients, which was a rarely published area, were revealed.

Although comprehensive database searches and review of national and international websites were conducted to collect the estimates of epidemiology data, little data were retrieved on la/mUC patients, indicating future studies based on large databases or national cancer registry data are required in China.

A variety of treatment options were observed from the search, while platinum‐based chemotherapy was identified to be the major treatment for la/mUC patients in China. The treatment outcomes of first‐line platinum‐based chemotherapy showing the ORR ranged from 41.2% to 77.51% and median OS ranged from 13.5 to 18 months.^32^, ^33^, ^34^, ^35^, ^36^, ^37^, ^38^, ^39^, ^40^, ^41^, ^42^ For first‐line standard therapy, international and national guidelines recommended gemcitabine plus cisplatin for cisplatin‐eligible patients, and gemcitabine plus carboplatin for the cisplatin‐ineligible patients.^1^, ^25^, ^26^ This review, however, identified four studies that did not administer platinum‐based chemotherapy as the front‐line therapy, including PD‐1/L1 in real‐world settings and non‐platinum‐based chemotherapy in clinical trials. Based on our findings, clarification on the rationale for not choosing platinum‐based chemotherapy was not often reported. Only one RCT explained that GE therapy was chosen over GC therapy as first‐line treatment, due to better tolerability and safety profiles for elderly patients.^53^ A real‐world study published in American Society of Clinical Oncology (ASCO) 2022 revealed that 39% of cisplatin‐eligible patients received PD‐1/L1 or other therapies (excluding GC, MVAC, and carboplatin + gemcitabine) as first‐line treatment.^75^ Our findings also identified that there were patients with metastatic bladder cancer who refused to initiate systemic chemotherapy, received percutaneous cryotherapy,^67^ which was not mentioned as a recommendation therapy in multiple guidelines,^1^, ^25^, ^26^ indicating that there are other considerations on safety and patient preference in addition to cisplatin‐eligibility for treatment decisions in the real‐world setting.

Most included studies focused on immunotherapy published from 2020 to 2022, implying an increasing investigation in ICIs since the first ICI indicated for la/mUC approved in China. For treatment‐naive and treatment‐experienced patients, ORR ranged from 24% to 54.5% and median OS ranged from 9.8 to 20.53 months.^15^, ^61^, ^62^, ^64^, ^65^ One currently published real‐world retrospective study among patients receiving different line of therapy, revealed an ORR of 28% in patients receiving PD‐1 inhibitors or PD‐1 inhibitors plus chemotherapy.^76^ However, given the reported outcomes were from studies with different sample size, inclusion of population with mixed treatment sequences and study designs, there is an apparent heterogeneity existing in this wide range of results and should be interpreted with caution. As for treatment‐naive patients, median PFS was significantly better in patients receiving PD‐1 plus chemotherapy than chemotherapy,^63^ while no significantly prolonged OS was observed. Same results were shown from the global, phase III multicenter IMvigor130 trial.^77^ Furthermore, another two global, phase III studies demonstrated that addition of ICI to chemotherapy did not significantly improve the efficacy outcomes in untreated patients.^10^, ^78^ Hence, further studies need to be conducted to validate whether the combination of immunotherapy and chemotherapy can improve clinical efficacy for untreated patients.

It was found that a substantial proportion of la/mUC patients cannot experience durable responses to ICIs,^79^, ^80^, ^81^ and treatment options are limited for patients who were refractory to platinum‐based chemotherapy and immunotherapy. Targeted therapies provided alternative and promising options for those patients, which associated with molecular alterations in various molecular subtypes, including PD‐L1 status, FGFR‐3 expression, HER2 expression, Nectin‐4 expression, and Trop‐2 expression.^11^, ^82^, ^83^, ^84^

Published reviews possessed different opinions regarding PD‐L1 status as a response biomarker of ICIs.^19^, ^85^ One review found that ICIs were associated with survival benefit compared with standard chemotherapy in PD‐L1 positive patients, while no benefit was observed in patients with PD‐L1 negative status in the first‐line setting.^85^ Another review suggested contrary findings based on trials conducted in untreated or previously treated patients.^19^ Inconclusive results may be associated with the use of different antibodies, evaluation of different compartments, that is tumor cells, immune cells or both via distinct scoring systems, and different cutoff value in definitions of PD‐L1 positive patients in studies.^25^, ^85^ Thus, more reliable biomarker is needed to improve outcomes for selected patients.

FGFR alterations have been detected in a wide variety of cancers, while the highest frequency was observed in UC, presenting in 17.9% to 32% among all cancer patients with FGFR alterations.^86^, ^87^ Within the FGFR family, FGFR3 alterations showed the highest prevalence,^87^ which is consistent with our findings.^68^, ^69^, ^70^, ^71^, ^72^ It is also worth noting that the frequency of FGFR3 alteration were generally lower among Chinese UC patients compared to the western patients based on included studies.^68^, ^72^, ^73^ In terms of FGFR3 alteration proportion among BC and UTUC patients, no significant difference was found among Chinese patients.^72^, ^73^ In contrast, significantly higher frequency was identified in UTUC compared to BC among western population,^73^ which was consistent with a previously conducted large cohort study.^88^ EAU guideline recommends FGFR3 testing to facilitate second‐line treatment choices of FGFR inhibitors for mUC patients.^25^ Erdafitinib, an FGFR inhibitor, was granted the accelerated approval by FDA. Its trial showed an ORR of 40% and a median OS of 13.8 months in chemotherapy‐experienced patients, of which some also have received ICIs.^89^ In addition, erdafitinib is also recommended by NCCN and CSCO guidelines as a second‐line and subsequent‐line therapy option for patients who have already received both a platinum‐containing therapy and a ICI.^1^, ^26^

Several ADCs were generated, including RC48‐ADC, enfortumab vedotin, and sacituzumab govitecan. RC48‐ADC, a HER2‐targeting ADC, was approved in China for HER2 overexpression la/mUC patients who failed chemotherapy, in which HER2 overexpression is defined as HER2 IHC2+ or 3+.^90^ Based on the clinical pathological expert consensus on HER2 testing in UC in China, HER2 testing is highly recommended for la/mUC patients to select potential patients who can benefit from HER2‐ADC.^91^ Testing results might be varied across testing methods due to their different sensitivity and specificity rates, at the same time the detection process and results interpretation have not achieved a global consensus yet.^91^ Enfortumab vedotin (EV), targeting Nectin‐4, was granted accelerated approval by the FDA and EMA for mUC patients who had previously received both platinum‐based chemotherapy regimen and immunotherapy,^92^, ^93^ and Sacituzumab govitecan which recognizes Trop‐2‐expressing cancer cells has received accelerated FDA approval for mUC patients with prior platinum and immunotherapy pretreatment.^84^, ^94^

Admittedly, this study has several limitations. First, quality assessment of included studies was not conducted due to the nature of comprehensive inclusion for literature diversity from scoping review study. Second, comparisons of the efficacy and safety across studies were difficult to be made because of differences in study design, data reporting such as missingness in LOT information. Third, 13 studies included less than 50 patients and 31 studies were conducted more than five years ago, which may not be reflective to current treatment patterns. In addition, since no epidemiological study meeting the inclusion criteria was identified, relevant websites have been searched, which reported the general epidemiology data for bladder cancer, while data on urothelial carcinoma specifically were not available.

## 5 CONCLUSION

Despite chemotherapy has remained a key role in the treatment option for Chinese la/mUC patients for decades, novel treatments of ICIs, targeted therapy, and ADCs are rapidly evolving, with a handful of agents that demonstrated clinical promise have obtained approval. Beyond the established standards integrated in multiple guidelines, standardization of treatments for la/mUC patients might be a solution to improve the treatment effectiveness and advance the current treatment landscape. Furthermore, pioneering large research in real‐world setting is encouraged given the limited real‐world evidence in la/mUC patients, and particularly among Chinese population. With the heterogenous performance of gene alterations across different patients, future study could be promoted to better understand the molecular biomarkers and pathways in la/mUC patients, and further explore potential precise therapies for specific molecular subtypes.

## AUTHOR CONTRIBUTIONS

**Wang He:** Conceptualization (lead); methodology (lead); supervision (equal); writing – original draft (equal); writing – review and editing (lead). **Changhao Chen:** Formal analysis (equal); investigation (equal); methodology (equal); validation (equal); visualization (equal); writing – original draft (equal). **Tianxin Lin:** Supervision (equal); writing – original draft (equal); writing – review and editing (equal). **Qian Xu:** Investigation (equal); validation (equal); writing – original draft (equal); writing – review and editing (equal). **Chong Ye:** Data curation (lead); formal analysis (equal); funding acquisition (equal); investigation (equal); methodology (equal); project administration (equal); writing – original draft (equal). **Jieyi Du:** Funding acquisition (equal); project administration (equal); resources (equal); writing – original draft (equal). **Jian Huang:** Conceptualization (equal); project administration (equal); supervision (lead); writing – review and editing (equal).

## FUNDING INFORMATION

This work was supported by Xi'an Janssen Pharmaceutical Ltd. The authors were responsible for all content and editorial decisions, and received no honoraria related to the development of this publication.

## CONFLICT OF INTEREST STATEMENT

Qian Xu, Chong Ye, and Jieyi Du were employees of Xi'An Janssen Pharmaceutical Ltd during the study. Other authors declare no competing interests.

## ETHICS APPROVAL STATEMENT

Not applicable.

## PATIENT CONSENT STATEMENT

Not applicable.

## PERMISSION TO REPRODUCE MATERIAL FROM OTHER SOURCES

Not applicable.

## CLINICAL TRIAL REGISTRATION

Not applicable.

## Supporting information


Table S1‐S11

## Data Availability

This review was based on published literature, all of which is fully listed.
