# Supplementary material for: Epidemiology, treatments, and related biomarkers of locally advanced or metastatic urothelial carcinoma in Chinese population: A scoping review
Source: Cancer Med. 2023 Jun 30;12(14):15384–403. doi: 10.1002/cam4.6112 (PMC10417093; doi:10.1002/cam4.6112)
Supplement: Supplementary file 1 — Table S1‐S11 [file CAM4-12-15384-s001.docx]

**List of Supplementary Information**

[Table 1 Population, concept, and context (PCC) framework 1](#_Toc120033829)

[Table 2 Search strategy for Web of Science 1](#_Toc120033830)

[Table 3 Search strategy for PubMed 3](#_Toc120033831)

[Table 4 Search strategy for Embase 5](#_Toc120033832)

[Table 5 Search strategy for CNKI 7](#_Toc120033833)

[Table 6 Search strategy for WANFANG 8](#_Toc120033834)

[Table 7 Search strategy for additional search of FGFR target in Web of Science 9](#_Toc120033835)

[Table 8 Search strategy for additional search of FGFR target in PubMed 10](#_Toc120033836)

[Table 9 Search strategy for additional search of FGFR target in Embase 12](#_Toc120033837)

[Table 10 Extraction Table Template for treatment options and associated efficacy and safety 14](#_Toc120033838)

[Table 11 Extraction Table Template for treatment-related biomarkers 15](#_Toc120033839)

Table 1 Population, concept, and context (PCC) framework

| **Element** | **Content** |
| --- | --- |
| Population | - Patients with locally advanced or metastatic urothelial carcinoma |
| Concept | - Epidemiology - Existing treatment options and associated efficacy and safety - Treatment-related biomarkers |
| Context | - Patients in mainland China, Taiwan, Hong Kong, Macau - Language: English and Chinese - Time limit: January 1, 2011–March 18, 2022 |

Table 2 Search strategy for Web of Science

| **Search** | **Category** | **Query** | **Number of records found** |
| --- | --- | --- | --- |
| 1 | Disease | TS=(("Urinary Bladder” or “urinary” OR “urothelial” or “urothelial cell” or “transitional cell” or “bladder” or “urogenital tract” or “upper tract urothelial” or “ureteral” or “urologic” or “renal pelvis” or “urethra*” or “Urothelium”) and (tumor* OR tumour* OR neoplasm* OR cancer OR carcinoma*))) AND TS=(“stage 3” OR “stage iii” OR “stage 3a” OR “stage iiia” OR “stage 3b” OR “stage iiib” OR “stage 4” OR “stage iv” OR metasta* OR advanc* OR relap* recurrence* OR refract* OR late-stage* OR “late stage*” OR inoperable OR “locally advanced” OR “locally-advanced”)) | 120,967 |
| 2 | Database | NOT (SILOID==("MEDLINE") | 33,601 |
| 3 | Publication year | 2011-2022 | 19,075 |
| 4 | Language | English | 17,805 |
| 5 | Document types | DT= ("ABSTRACT" OR "ARTICLE") | 12,550 |
| 6 | Conference titles | exclude all | 2,984 |
| 7 | Countries/regions | #6 and (PEOPLES R CHINA or CHINA PEOPLES REPUBLIC OF CHINA or TAIWAN) | 494 |
| 8 |  | #6 and (TS=("China"OR "Chinese"OR "Taiwan"OR "Taiwanese" OR "Hong Kong" OR "HongKonger" OR "Macao" OR "Macau" OR "Macanese")) | 78 |
| 9 |  | #7 OR #8 | 494 |
| 10 | Epidemiology / patient characteristics | #9 AND TS= (Incidence OR prevalence OR epidemiology OR morbidity OR mortality) | 89 |
| 11 | Existing treatment options / efficacy / effectiveness / safety | #9 AND TS= ("Complete response" OR "Partial response" OR "Objective response rate" OR "Progression free survival" OR "Duration of response" OR "Overall survival" OR "Response rate" OR "cancer specific survival" OR "disease free survival" OR "Distant metastasis free survival" OR "recurrence free survival" OR "event free survival" OR "disease recurrence" OR "disease progression" OR "adverse event*" OR safety or toxicity) | 125 |
| 12 | Molecular diagnosis / biomarkers | #9 AND TS= (FISH OR "fluorescence in situ hybrid*" OR "NGS" OR "PCR" OR biomarker* OR "molecular diagnos*" OR "mutation*" OR "mutate*"OR "fusion*" OR "fuse" OR "fused" or "fusing" OR "amplification*" OR "amplicate*" OR "translocation*" OR "translocat*") | 146 |

Table 3 Search strategy for PubMed

| **Search** | **Category** | **Query** | **Number of records found** |
| --- | --- | --- | --- |
| 1 | Disease | ("Urinary Bladder"[Title/Abstract] OR "urinary"[Title/Abstract] OR "urothelial"[Title/Abstract] OR "urothelial cell"[Title/Abstract] OR "transitional cell"[Title/Abstract] OR "bladder"[Title/Abstract] OR "urogenital tract"[Title/Abstract] OR "upper tract urothelial"[Title/Abstract] OR "ureteral"[Title/Abstract] OR "urologic"[Title/Abstract] OR "renal pelvis"[Title/Abstract] OR "urethra*"[Title/Abstract] OR "Urothelium"[Title/Abstract]) AND ("tumor*"[Title/Abstract] OR "tumour*"[Title/Abstract] OR "neoplasm*"[Title/Abstract] OR "cancer"[Title/Abstract] OR "carcinoma*"[Title/Abstract]) | 121,404 |
| 2 | Disease | AND (“stage 3”[Title/Abstract] OR “stage iii”[Title/Abstract] OR “stage 3a”[Title/Abstract] OR “stage iiia”[Title/Abstract] OR “stage 3b”[Title/Abstract] OR “stage iiib”[Title/Abstract] OR “stage 4”[Title/Abstract] OR “stage iv”[Title/Abstract] OR metasta*[Title/Abstract] OR advanc*[Title/Abstract] OR relap*[Title/Abstract] OR recurrence*[Title/Abstract] OR refract*[Title/Abstract] OR late-stage*[Title/Abstract] OR “late stage*”OR inoperable[Title/Abstract] OR “locally advanced”[Title/Abstract] OR “locally-advanced”[Title/Abstract]) | 38,753 |
| 3 | Countries/regions | AND ("China"[All Fields] OR "Chinese"[All Fields] OR "Taiwan"[All Fields] OR "Taiwanese"[All Fields] OR "Hong Kong"[All Fields] OR "Macao"[All Fields] OR "Macau"[All Fields] OR "Macanese"[All Fields]) | 4,594 |
| 4 | Publication type | NOT ("Biography"[Publication Type] OR "Case Reports"[Publication Type] OR "Clinical Trial Protocol"[Publication Type] OR "Comment"[Publication Type]OR "Congress"[Publication Type] OR "Editorial"[Publication Type] OR "Electronic Supplementary Materials"[Publication Type] OR “Introductory Journal Article”[Publication Type] OR "Guideline"[Publication Type] OR "Interactive Tutorial"[Publication Type] OR "Letter"[Publication Type] OR "Meta-Analysis"[Publication Type] OR "Practice Guideline"[Publication Type] OR "Retracted Publication"[Publication Type] OR "Review"[Publication Type] OR "Systematic Review" OR "Video-Audio Media"[Publication Type]) | 3,621 |
| 5 | Publication year | AND 2011-2022 | 3,067 |
| 6 | language | AND English | 2,849 |
| 7 | Epidemiology / patient characteristics | #6 AND (Incidence[Title/Abstract] OR prevalence[Title/Abstract] OR epidemiology[Title/Abstract] OR morbidity[Title/Abstract] OR mortality[Title/Abstract]) | 402 |
| 8 | Existing treatment options / efficacy / effectiveness / safety | #6 AND (Complete response[Title/Abstract] OR "Partial response"[Title/Abstract] OR "Objective response rate"[Title/Abstract] OR "Progression free survival"[Title/Abstract] OR "Duration of response"[Title/Abstract] OR "Overall survival"[Title/Abstract] OR "Response rate"[Title/Abstract] OR "cancer specific survival"[Title/Abstract] OR "disease free survival"[Title/Abstract] OR "Distant metastasis free survival"[Title/Abstract] OR "recurrence free survival"[Title/Abstract] OR "event free survival"[Title/Abstract] OR "disease recurrence"[Title/Abstract] OR "disease progression"[Title/Abstract] OR "adverse event*"[Title/Abstract] OR safety[Title/Abstract] OR toxicity[Title/Abstract]) | 1,044 |
| 9 | Molecular diagnosis / biomarkers | #6 AND ("FISH"[Title/Abstract] OR "fluorescence in situ hybrid*"[Title/Abstract] OR "NGS"[Title/Abstract] OR "PCR"[Title/Abstract] OR biomarker*[Title/Abstract] OR "molecular diagnos*"[Title/Abstract] OR "mutation*"[Title/Abstract] OR "mutate*"OR "fusion*"[Title/Abstract] OR "fuse"[Title/Abstract] OR "fused"[Title/Abstract] OR "fusing"[Title/Abstract] OR "amplification*"[Title/Abstract] OR "amplicate*"[Title/Abstract] OR "translocation*"[Title/Abstract] OR "translocat*"[Title/Abstract]) | 917 |

Table 4 Search strategy for Embase

| **Search** | **Category** | **Query** | **Number of records found** |
| --- | --- | --- | --- |
| 1a | Disease | ((('urinary bladder':ab,ti OR urinary:ab,ti OR urothelial:ab,ti) OR ('urothelial cell':ab,ti OR 'transitional cell':ab,ti OR bladder:ab,ti OR 'urogenital tract':ab,ti OR 'upper tract urothelial':ab,ti) OR (ureteral:ab,ti OR urologic:ab,ti OR 'renal pelvis':ab,ti OR 'urethra*':ab,ti OR urothelium:ab,ti)) AND ((tumor*:ab,ti OR tumour*:ab,ti) OR (neoplasm*:ab,ti OR cancer:ab,ti OR carcinoma*:ab,ti))) | 177,311 |
| 1b | Disease | stage 3':ab,ti OR 'stage iii':ab,ti OR 'stage 3a':ab,ti OR 'stage iiia':ab,ti OR 'stage 3b':ab,ti OR 'stage iiib':ab,ti OR 'stage 4':ab,ti OR 'stage iv':ab,ti OR 'advanc*':ab,ti OR 'metasta*':ab,ti OR 'relap*':ab,ti OR 'recurrence*':ab,ti OR 'refract*':ab,ti OR 'late-stage*':ab,ti OR 'late stage':ab,ti OR inoperable:ab,ti OR 'locally advanced':ab,ti OR 'locally-advanced':ab,ti | 63,352 |
| 2 | Publication year | AND 2011-2022 | 40,939 |
| 3 | Countries/regions | AND ('China' OR 'Chinese' OR 'Taiwan' OR 'Taiwanese' OR 'Hong Kong' OR 'HongKonger' OR 'Macao' OR 'Macau' OR 'Macanese') | 5,652 |
| 4 | Data source | AND (([embase]/lim NOT ([embase]/lim AND [medline]/lim)) | 2,743 |
| 5 | Language | AND English | 2,456 |
| 6 | Publication type | NOT ('conference abstract'/it OR 'conference paper'/it OR 'conference review'/it OR 'data paper'/it OR 'editorial'/it OR 'erratum'/it OR 'letter'/it OR 'note'/it OR 'review'/it OR 'short survey'/it) | 1,053 |
| 7 | Study type | 'AND 'case control study'/de OR 'clinical article'/de OR 'clinical trial'/de OR 'cohort analysis'/de OR 'comparative effectiveness'/de OR 'comparative study'/de OR 'controlled study'/de OR 'diagnostic test accuracy study'/de OR 'major clinical study'/de OR 'multicenter study'/de OR 'observational study'/de OR 'phase 1 clinical trial'/de OR 'phase 2 clinical trial'/de OR 'phase 3 clinical trial'/de OR 'prospective study'/de OR 'randomized controlled trial'/de OR 'retrospective study'/de OR 'longitudinal study'/de OR 'cross-sectional study'/de OR 'phase 4 clinical trial'/de OR 'clinical study'/de OR 'control group'/de OR 'controlled clinical trial'/de | 866 |
| 8 | Species | AND humans | 846 |
| 9 | Epidemiology | #8 AND (incidence:ab,ti OR prevalence:ab,ti OR epidemiology:ab,ti OR morbidity:ab,ti OR mortality:ab,ti) | 101 |
| 10 | Existing treatment options / efficacy / effectiveness / safety | #8 AND ('complete response':ab,ti OR 'partial response':ab,ti OR 'objective response rate':ab,ti OR 'progression free survival':ab,ti OR 'duration of response':ab,ti OR 'overall survival':ab,ti OR 'response rate':ab,ti OR 'cancer specific survival':ab,ti OR 'disease free survival':ab,ti OR 'distant metastasis free survival':ab,ti OR 'recurrence free survival':ab,ti OR 'event free survival':ab,ti OR 'disease recurrence':ab,ti OR 'disease progression':ab,ti OR 'adverse event*':ab,ti OR safety:ab,ti OR toxicity:ab,ti) | 327 |
| 11 | Molecular diagnosis / biomarkers | #8 AND (fish:ab,ti OR 'fluorescence in situ hybrid*':ab,ti OR ngs:ab,ti OR pcr:ab,ti OR 'bimomarker*':ab,ti OR 'molecular diagnos*':ab,ti OR 'mutation*' OR 'mutate*':ab,ti OR 'fusion*':ab,ti OR fuse:ab,ti OR fused:ab,ti OR fusing:ab,ti OR 'amplification*':ab,ti OR 'amplicate*':ab,ti OR 'translocation*':ab,ti OR 'tarnslocat*':ab,ti) | 177 |

Table 5 Search strategy for CNKI

| **Search** | **Category** | **Query** | **Number of records found** |
| --- | --- | --- | --- |
| 1 | Disease | TKA= ("尿路上皮癌" + "尿路上皮肿瘤" + "膀胱尿路上皮癌" + "膀胱尿路上皮肿瘤" + "上尿路尿路上皮癌" + "上尿路尿路上皮肿瘤" + "肾盂癌" + "肾盂肿瘤" + "输尿管肿瘤" + "输尿管癌" + “膀胱癌” + “膀胱肿瘤”) AND TKA= ("转移" + "局部晚期" + "局部进展" + "晚期" + "复发") | 10,461 |
| 2 | Date | AND Date:2011-2022 | 6,246 |
|  | Publication type | AND 学术期刊 | 3,516 |
| 3 | Database | AND 核心期刊 | 700 |
| 4 | Language | AND 中文 | 700 |
| 5 | Publication type | NOT TI= ("1例" + "一例" + "文献复习") | 651 |
| 6 | Publication type | NOT TKA= ("综述" + "系统综述" + "Meta分析" + "荟萃分析" + "病例报告" + "病历报告") | 592 |
| 7 | Epidemiology | TKA= (“流行病" + "发病率" + "患病率" + "存活率" + "死亡率") | 41 |
| 8 | Existing treatment options / efficacy / effectiveness / safety | TKA= ("完全缓解" + "部分缓解" + "客观缓解率" + "无进展生存期" + "缓解持续时间" + "总生存期" + "癌症特应性生存率" + "无事件生存" + "疾病进展" + "疾病复发" + "不良反应" + "不良事件" + "安全性" + "毒性" + "治疗" + "化疗" + "诊疗" + "疗效" + "效果" + "缓解") | 352 |
| 9 | Molecular diagnosis / biomarkers | TKA= ("FISH" + "NGS"+ "PCR" + "突变" + "融合" + "扩增" + "易位" + "基因" + "分子" + "诊断" + "检测" + "标记物" + "标志物"） | 404 |

***CNKI is a Chinese database.**

Table 6 Search strategy for WANFANG

| **Search** | **Category** | **Query** | **Number of records found** |
| --- | --- | --- | --- |
| 1 | Publication type | 期刊论文 |  |
| 2 | Disease | 主题:("尿路上皮癌" OR "尿路上皮肿瘤" OR "膀胱尿路上皮癌" OR "膀胱尿路上皮肿瘤" OR "上尿路尿路上皮癌" OR "上尿路尿路上皮肿瘤" OR "肾盂癌" OR "肾盂肿瘤" OR "输尿管肿瘤" OR "输尿管癌" OR “膀胱癌” OR “膀胱肿瘤”) and 主题:("转移" OR "局部晚期" OR "局部进展" OR "晚期") | 4,390 |
| 3 | Date | AND 2011-2021 | 2,581 |
| 4 | Language | AND中文 | 2,570 |
| 5 | Publication quality | AND核心期刊 | 1,905 |
| 6 | Publication type | NOT题名:("1例" OR "一例" OR "文献复习") | 1,755 |
| 7 | Publication type | NOT 主题:("综述" OR "系统综述" OR "Meta分析" OR "荟萃分析" OR "病例报告" OR "病历报告") | 1,547 |
| 8 | Epidemiology | AND 主题:("流行病" OR "发病率" OR "患病率" OR "存活率" OR "死亡率") | 117 |
| 9 | Existing treatment options / efficacy / effectiveness / safety | AND 主题: ("完全缓解" OR "部分缓解" OR "客观缓解率" OR "无进展生存期" OR "缓解持续时间" OR "总生存期" OR "癌症特应性生存率" OR "无事件生存" OR "疾病进展" OR "疾病复发" OR "不良反应" OR "不良事件" OR "安全性" OR "毒性" OR "治疗" OR "化疗" OR "诊疗" OR "疗效" OR "效果" OR "缓解") | 948 |
| 10 | Molecular diagnosis / biomarkers / pathological diagnosis | AND 主题:("FISH" OR "NGS" OR "PCR" OR "突变" OR "融合" OR "扩增" OR "易位" OR "基因" OR "分子" OR "诊断" OR "检测" OR "标记物" OR "标志物") | 950 |

***WANGFANG is a Chinese database.**

Table 7 Search strategy for additional search of FGFR target in Web of Science

| **Search** | **Category** | **Query** | **Number of records found** |
| --- | --- | --- | --- |
| 1 | Disease | TS=(("Urinary Bladder” or “urinary” OR “urothelial” or “urothelial cell” or “transitional cell” or “bladder” or “urogenital tract” or “upper tract urothelial” or “ureteral” or “urologic” or “renal pelvis” or “urethra*” or “Urothelium”) and (tumor* OR tumour* OR neoplasm* OR cancer OR carcinoma*))) | 632,201 |
| 2 | Database | NOT (SILOID==("MEDLINE") | 257,245 |
| 3 | Publication year | 2011-2022 | 114,506 |
| 4 | Language | English | 112,494 |
| 5 | Document types | DT= ("ABSTRACT" OR "ARTICLE") | 75,209 |
| 6 | Conference titles | exclude all | 23,009 |
| 7 | Countries/regions | #6 and (PEOPLES R CHINA or CHINA PEOPLES REPUBLIC OF CHINA or TAIWAN) | 2,797 |
| 8 |  | #6 and (TS=("China"OR "Chinese"OR "Taiwan"OR "Taiwanese" OR "Hong Kong" OR "HongKonger" OR "Macao" OR "Macau" OR "Macanese")) | 680 |
| 9 |  | #7 OR #8 | 2,970 |
| 10 | FGFR Target | #9 AND TS= (FGFR* OR "Fibroblast growth factor receptor" OR Ponatinib OR Nintedanib OR Dovitinib OR Erdafitinib OR Alofanib OR Lucitanib OR Infigratinib OR Rogaratinib OR AZD4547 OR CH5183284 OR TAS120 OR Pemigatinib OR derazantinib) | 15 |

Table 8 Search strategy for additional search of FGFR target in PubMed

| **Search** | **Category** | **Query** | **Number of records found** |
| --- | --- | --- | --- |
| 1 | Disease | ("Urinary Bladder"[Title/Abstract] OR "urinary"[Title/Abstract] OR "urothelial"[Title/Abstract] OR "urothelial cell"[Title/Abstract] OR "transitional cell"[Title/Abstract] OR "bladder"[Title/Abstract] OR "urogenital tract"[Title/Abstract] OR "upper tract urothelial"[Title/Abstract] OR "ureteral"[Title/Abstract] OR "urologic"[Title/Abstract] OR "renal pelvis"[Title/Abstract] OR "urethra*"[Title/Abstract] OR "Urothelium"[Title/Abstract]) AND ("tumor*"[Title/Abstract] OR "tumour*"[Title/Abstract] OR "neoplasm*"[Title/Abstract] OR "cancer"[Title/Abstract] OR "carcinoma*"[Title/Abstract]) | 121,404 |
| 2 | Countries/regions | AND ("China"[All Fields] OR "Chinese"[All Fields] OR "Taiwan"[All Fields] OR "Taiwanese"[All Fields] OR "Hong Kong"[All Fields] OR "Macao"[All Fields] OR "Macau"[All Fields] OR "Macanese"[All Fields]) | 14,248 |
| 3 | Publication type | NOT ("Biography"[Publication Type] OR "Case Reports"[Publication Type] OR "Clinical Trial Protocol"[Publication Type] OR "Comment"[Publication Type]OR "Congress"[Publication Type] OR "Editorial"[Publication Type] OR "Electronic Supplementary Materials"[Publication Type] OR “Introductory Journal Article”[Publication Type] OR "Guideline"[Publication Type] OR "Interactive Tutorial"[Publication Type] OR "Letter"[Publication Type] OR "Meta-Analysis"[Publication Type] OR "Practice Guideline"[Publication Type] OR "Retracted Publication"[Publication Type] OR "Review"[Publication Type] OR "Systematic Review" OR "Video-Audio Media"[Publication Type]) | 11,582 |
| 4 | Publication year | AND 2011-2022 | 9,387 |
| 5 | language | AND English | 8,835 |
| 6 | FGFR Target | AND (FGFR*[Title/Abstract] OR "Fibroblast growth factor receptor"[Title/Abstract] OR Ponatinib[Title/Abstract] OR Nintedanib[Title/Abstract] OR Dovitinib[Title/Abstract] OR Erdafitinib[Title/Abstract] OR Alofanib[Title/Abstract] OR Lucitanib[Title/Abstract] OR Infigratinib[Title/Abstract] OR Rogaratinib[Title/Abstract] OR AZD4547[Title/Abstract] OR CH5183284[Title/Abstract] OR TAS120[Title/Abstract] OR Pemigatinib[Title/Abstract] OR derazantinib[Title/Abstract]) | 96 |

Table 9 Search strategy for additional search of FGFR target in Embase

| **Search** | **Category** | **Query** | **Number of records found** |
| --- | --- | --- | --- |
| 1 | Disease | ((('urinary bladder':ab,ti OR urinary:ab,ti OR urothelial:ab,ti) OR ('urothelial cell':ab,ti OR 'transitional cell':ab,ti OR bladder:ab,ti OR 'urogenital tract':ab,ti OR 'upper tract urothelial':ab,ti) OR (ureteral:ab,ti OR urologic:ab,ti OR 'renal pelvis':ab,ti OR 'urethra*':ab,ti OR urothelium:ab,ti)) AND ((tumor*:ab,ti OR tumour*:ab,ti) OR (neoplasm*:ab,ti OR cancer:ab,ti OR carcinoma*:ab,ti))) | 181,515 |
| 2 | Publication year | AND 2011-2022 | 104,978 |
| 3 | Countries/regions | AND ('China' OR 'Chinese' OR 'Taiwan' OR 'Taiwanese' OR 'Hong Kong' OR 'HongKonger' OR 'Macao' OR 'Macau' OR 'Macanese' [All fields]) | 16,077 |
| 4 | Data source | AND (([embase]/lim NOT ([embase]/lim AND [medline]/lim)) | 7,286 |
| 5 | Language | AND English | 6,525 |
| 6 | Publication type | NOT ('conference abstract'/it OR 'conference paper'/it OR 'conference review'/it OR 'data paper'/it OR 'editorial'/it OR 'erratum'/it OR 'letter'/it OR 'note'/it OR 'review'/it OR 'short survey'/it) | 3,016 |
| 7 | Study type | 'AND 'case control study'/de OR 'clinical article'/de OR 'clinical trial'/de OR 'cohort analysis'/de OR 'comparative effectiveness'/de OR 'comparative study'/de OR 'controlled study'/de OR 'diagnostic test accuracy study'/de OR 'major clinical study'/de OR 'multicenter study'/de OR 'observational study'/de OR 'phase 1 clinical trial'/de OR 'phase 2 clinical trial'/de OR 'phase 3 clinical trial'/de OR 'prospective study'/de OR 'randomized controlled trial'/de OR 'retrospective study'/de OR 'longitudinal study'/de OR 'cross-sectional study'/de OR 'phase 4 clinical trial'/de OR 'clinical study'/de OR 'control group'/de OR 'controlled clinical trial'/de | 2,515 |
| 8 | Species | AND humans | 2,378 |
| 9 | FGFR Target | #8 AND (fgfr*:ab,ti OR 'fibroblast growth factor receptor':ab,ti OR ponatinib:ab,ti OR nintedanib:ab,ti OR dovitinib:ab,ti OR erdafitinib:ab,ti OR alofanib:ab,ti OR lucitanib:ab,ti OR infigratinib:ab,ti OR rogaratinib:ab,ti OR azd4547:ab,ti OR ch5183284:ab,ti OR tas120:ab,ti OR pemigatinib:ab,ti OR derazantinib:ab,ti) | 25 |

Table 10 Extraction Table Template for treatment options and associated efficacy and safety

| **Publication details** | **Study overview** | **Patient characteristics** |
| --- | --- | --- |
| - Citation ID - Title - Author - Abstract - Publication year | - Study design - Study type - Location / Region - Study duration / period - Follow-up duration - Target Population - Diagnosis - Leading site - Study objective - Primary outcome - Secondary outcomes - Study Conclusion - Sample size - Number of intention-to-treat   population   - Number of modified   intention-to-treat population | - Group - Number of populations - Age - Male - Prior treatment - Line of therapy - ECOG - Creatinine clearance rate - Detection of target   expression   - Tumor stage - Lymph node status N+ - Metastases - Lesion - Upper tract |

| **Efficacy outcomes of treatment** | **Safety outcomes of treatment** |
| --- | --- |
| - Treatment Pattern - Type of endpoint - Outcome - Number of populations - Outcome definition - Timepoint of evaluation - Number of outcome events - Outcome results - Lower Confidence Interval - Upper Confidence Interval - Standard Deviation - P-value | - Type of endpoint - Adverse event - Grade of Adverse event - Number of populations - Number of events/safety analysis set |

Table 11 Extraction Table Template for treatment-related biomarkers

| **Publication details** | **Study overview** | **Patient characteristics** |
| --- | --- | --- |
| - Citation ID - Title - Author - Abstract - Publication year | - Study design - Study type - Location / Region - Target Population - Leading site - Study objective - Study Conclusion - Subgroup - Sample size | - Group - Number of populations - Age - Male - Prior treatment - Line of therapy - ECOG - Creatinine clearance rate - Detection of target   expression   - Tumor stage - Lymph node status N+ - Metastases - Lesion - Upper tract |

| **Detection** | **Gene mutation /amplification / fusion; Protein expression** |
| --- | --- |
| - Detection method - Tumor location - Sensitivity - Specificity | - Sample types - Sample size - Alteration type - Alteration gene / expression protein - Alteration number of the gene / expression   number   - Alteration rate of the gene / expression rate   of protein   - Gene alteration region |
